# Advancing Thermoset Technology: 4R Materials with Unchanged Mechanical Properties and Enhanced Sustainability Through Repellency, Recyclability, Reprocessability, and Repairability

**DOI:** 10.3390/polym17233147

**Published:** 2025-11-26

**Authors:** Aratz Genua, Nagore Indakoetxea, Edurne Elorza, Jagoba Iturri, Paula Fanlo, Elena Jubete, Hans-J. Grande, Ignacio Garcia

**Affiliations:** 1 CIDETEC, Basque Research and Technology Alliance (BRTA), P° Miramón 196, 20014 Donostia-San Sebastian, Spain; agenua@cidetec.es (A.G.); ejubete@cidetec.es (E.J.);; 2 Advanced Polymers and Materials: Physics, Chemistry and Technology Department, University of the Basque Country (UPV/EHU), Avda. Tolosa 72, 20018 Donostia-San Sebastian, Spain

**Keywords:** omniphobic, repellent, repairable, reprocessable, recyclable, CANs

## Abstract

In this work, we present the development of a novel thermoset epoxy network that combines highly dynamic characteristics (reprocessability, reparability, and recyclability, 3R) with enhanced surface repellency. The conventional dynamic hardener of a 3R epoxy network was partially substituted with two different polydimethylsiloxane (PDMS) compounds at varying weight percentages and molecular weights, allowing us to systematically evaluate their influence on the material’s properties. Mechanical, thermal, and dynamic characteristics were retained, confirming that the substitution does not compromise the functional integrity of the network. Importantly, the surface properties were significantly improved, demonstrating enhanced repellency without affecting the core 3R functionality. A comparative analysis of the two PDMS variants revealed the influence of chemical structure and molecular weight on surface performance, providing insights into the design of multifunctional, sustainable epoxy networks.

## 1. Introduction

Epoxy thermosets are popular matrix materials for high-performance composites used in industries like aerospace, wind energy, and transportation due to their strength, heat resistance, and dimensional stability. However, their crosslinked structure limits recyclability and repairability, creating environmental and sustainability challenges [1]. To enhance the sustainability of the composite industry and ensure alignment with circular economy objectives, the implementation of new strategies is essential. Dynamic polymer networks, especially covalent adaptable networks (CANs), offer a promising solution by maintaining the strength of traditional thermosets while allowing reversible bond exchanges that enable repair, reshaping and recycling [2,3]. Incorporating CAN chemistry into epoxy-based composites therefore offers a pathway toward more sustainable materials, aligning the high-performance requirements of advanced industries with the principles of circularity [4,5,6].

To transform the polymer matrices into CANs that combine the structural rigidity of the thermosets with the adaptability of thermoplastics and, thus, can be easily recycled, we focus on incorporating dynamic covalent bonds into the epoxy thermosetting network [7,8,9,10,11]. Their crosslinked structure provides stability during use, while dynamic covalent bonds allow reversible bond exchanges, giving the material the ability to be reprocessed, repaired, and recycled. CANs are classified as associative or dissociative based on their bond exchange mechanism. Associative CANs maintain constant crosslink density by exchanging bonds without breaking the network, preserving mechanical strength while allowing reprocessing and repair. Dissociative CANs, on the other hand, temporarily break bonds, reducing crosslink density and viscosity during reprocessing. The chosen bond exchange mechanism strongly influences the material’s mechanical and reprocessing behavior. Within these categories, numerous dynamic bonds exist, giving rise to a wide variety of CANs with diverse properties and applications [12,13]. For instance, our research group has developed epoxy systems utilizing reversible aromatic disulfide linkages. These materials, incorporated into an epoxy matrix, enable the production of carbon fiber-reinforced composites that are reprocessable, repairable, and recyclable (3R) [14]. The aromatic disulfide crosslinks are easily introduced into the epoxy network using readily available amine-terminated hardeners, such as 4-aminophenyl disulfide (4-AFD).

Epoxy resins can be made more liquid repellent by adding materials with low surface free energy, which helps prevent liquids and stains from adhering to and remaining on the surface [15,16,17,18,19]. Most of these technologies rely on perfluorinated substances (PFAS) and solvent-based formulations (such as alcohols, ketones, and fluorinated solvents), which pose significant environmental and health risks. In this context, there is an urgent need to develop PFAS-free coatings that are more environmentally friendly and safer for human health [20,21].

Polydimethylsiloxane (PDMS) is an attractive PFAS alternative for high-performance coatings because it is non-toxic, transparent, flexible, and highly resistant to heat, chemicals, oxidation, and weathering, while also being naturally hydrophobic [22,23,24,25,26,27,28,29,30,31]. Several researchers have explored the integration of PDMS into epoxy systems [32,33,34]. Hyperbranched polysiloxanes, consisting of various siloxane units with different terminal substituent groups, have also been investigated [35,36,37]. Other modification strategies have been reported as well: addition of microcapsules (ionic PDMS oligomer core with a PMMS shell) to a commercial epoxy coating for improved corrosion resistance [38]; using a blend of functional acrylic polysiloxane polymer and liquid epoxy resin with common micron-sized inorganic particles, to develop a base coat suitable for a simple spray-applied omniphobic coating [39]; and blending OH-terminated PDMS and a rigid epoxy resin to improve hydrophobicity, mechanical properties, and corrosion resistance for marine applications [15,40].

Polysiloxane-modified CANs improve toughness, flexibility, and dynamic reconfigurability by enhancing filler dispersion and forming flexible crosslinks, resulting in mechanically robust, highly elastic materials [41]. However, issues like phase separation brought on by the siloxane and epoxy phases’ poor compatibility still have an impact on long-term stability and homogeneity. Rapid stress relaxation, self-healing, and recyclability have been demonstrated by studies introducing dual dynamic bonding mechanisms, such as transesterification and siloxane exchange [42]. Dynamic bond mechanisms in vitrimers, especially those combining siloxane and disulfide bonds, improve strength and relaxation properties but require high temperatures or catalysts and pose challenges in controlling reaction rates, reproducibility, and energy efficiency [43]. Overall, balancing performance, processability, and scalability is a key challenge for siloxane-based flexible epoxy thermosets, despite their potential for durable, reprocessable, and sustainable materials.

In this work, we present the development of a multifunctional epoxy matrix by combining the 3R technology and an omniphobic surface modification to create 4R materials: Repellent, Recyclable, Reprocessable, and Repairable. These innovative materials were produced through the addition of different PDMS molecules containing terminal NH_2_ functional groups, which covalently react with the epoxy matrix. The resulting modified resins exhibit improved surface properties—such as repellency—without compromising their 3R functionalities. Furthermore, the new surface properties remained unaltered after recycling, reprocessing, and repairing these materials, demonstrating the robustness and versatility of the 4R approach.

## 2. Materials and Methods

### 2.1. Materials

All chemicals were used as received without further purification. Regarding PDMS molecules with different structures and weight average molecular weights (*M*_w_), PDMS aminopropyl terminated *M*_w_ 850–900 g·mol^−1^, PDMS aminopropyl terminated *M*_w_ 3000 g·mol^−1^ and PDMS monoaminopropyl terminated *M*_w_ 6000 g·mol^−1^ were purchased from ABCR (Karlsruhe, Germany), while PDMS monoaminopropyl terminated *M*_w_ 2000 g·mol^−1^ was acquired from Gelest (Morrisville, PA, USA). *n*-Heptane (99%) was ordered from Scharlab (Sentmenat, Barcelona, Spain). Epikote™ Resin MGS™ RIMR 135 epoxy resin (epoxy equivalent weight (EEW) = 175 g·eq^−1^) was purchased from Hexion Specialty Chemicals Ibérica (Hernani, Spain) and 4-aminophenyl disulfide (4-AFD, 98%) was purchased from Molekula (Darlington, UK). Hexadecane (98%) was purchased from Sigma-Aldrich (Munich, Germany) and ultrapure water (<0.054 μS/cm) was obtained by using a Millipore (Burlington, MA, USA) Elix 3 water purification systems.

### 2.2. Blending Protocol

In order toto study the influence of the *M*_w_ and the number of functional groups of the PDMS on the final properties of the cured epoxy thermoset matrix, two different monoamine-terminated PDMS molecules (with *M*_w_ of 2000 g·mol^−1^ and 6000 g·mol^−1^, named MA2000 and MA6000, respectively) and two different diamine-terminated PDMS molecules (with *M*_w_ of 850 g·mol^−1^ and 3000 g·mol^−1^, named DA850 and DA3000, respectively) were studied. Table 1 shows the code name of the molecules that were investigated in this work and their main characteristics.

The ternary PDMS/epoxy/amine systems as well as the reference epoxy/amine system (all formulations shown in Tables S1–S4) were prepared using the following procedure: Epikote™ Resin MGS™ RIMR 135 epoxy was weighed and the corresponding PDMS amount was added at a different weight percentage (with respect to the epoxy, considering its EEW of 175 g·eq^−1^). The mixture was heated up to 60 °C with continuous stirring to decrease the viscosity and improve the homogeneity. Afterwards, 4-AFD, the dynamic hardener (previously melted at 80 °C), was added and mixed until obtaining a homogeneous blend. An excess of hardener was always employed (1.2 amino eq. per 1 epoxy eq., the amino content of the PDMS was also taken into consideration for these calculations). Aliphatic amines are more reactive than aromatic ones, so the excess of hardener permits on the one hand to introduce extra aromatic disulfide bonds in the system and, on the other hand, to have free amine groups, both of which increase the dynamism of the system [44]. The liquid mixture was then poured between two glass plates separated by a 2.5- or 1.5 mm rubber spacer to manually prepare flat laminates for further characterization (diameter of the rubber depending on the characterization that was carried out on the laminate). The curing cycle was determined by preliminary DSC studies and set as 1.5 h at 130 °C with a post-curing of 0.5 h at 150 °C. After curing, samples were slowly cooled down to room temperature and demolded. Subsequently, all the samples were washed with heptane by placing them in an ultrasonic bath for 5 min to remove the unreacted PDMS molecules. The complete reaction of PDMS was verified by washing the samples with heptane and evaluating their surface properties. If the surface properties remained unchanged after washing, this indicated that all PDMS had reacted and become integrated into the epoxy matrix, suggesting that the PDMS content in the mixture could be increased. Conversely, if the surface properties changed after washing, it would indicate that the PDMS had not fully reacted and was removed by the heptane wash. In such cases, the sample was rejected, and the PDMS content was not increased further.

### 2.3. Characterization Techniques

The wetting properties of the resulting materials were characterized in terms of static and dynamic contact angles and sliding angles using water and hexadecane as liquid probes; a Theta 200-Basic optical tensiometer from Biolin Scientific (Västra Frölunda, Sweden) was used to determine them. Droplets of solvents were placed on a minimum of three different areas of the surface. The static contact angles were recorded using the Laplace-Young fitting method. The sliding and static contact angles of the samples were measured using deionized water and hexadecane with a 10 µL droplet of liquid. In case of sliding angle measurements, the rate of tilt was 1°·s^−1^ and the tilt range was from 0° to 90°. All tests were carried out at room temperature.

The morphological characterization of the surface was carried out by ULTRA plus ZEISS FESEM (Oberkochen, Germany) field emission scanning electron microscope (FESEM). Elemental compositions were analyzed by energy dispersive X-ray spectroscopy (EDS) using an energy dispersive X-ray spectrometer installed with the scanning electron microscope. The fractured surfaces were prepared by immersing samples in liquid nitrogen.

Average surface roughness (Ra), roughness depth (Rz) and topography were measured with a LEICA DCM3D confocal microscopy from Leica Microsystems (Wetzlar, Germany). Data analysis was carried out using a Gaussian filter of 0.8 mm.

Thermal stability was measured by thermogravimetric analysis (TGA) using a TA Instruments TGA Q500 (New Castle, DE, USA). Measurements were performed from 25 °C to 700 °C at a heating rate of 10 °C·min^−1^ under a nitrogen atmosphere.

Thermal analysis was performed by differential scanning calorimetry (DSC) using a TA Instruments Discovery DSC 25 Auto (New Castle, DE, USA) over the temperature range 25–200 °C under a nitrogen atmosphere. A scan rate of 20 °C·min^−1^ was used for determining the glass transition temperature (*T_g_*) (which was obtained as the half-height of the heat flow step), while a scan rate of 1 °C·min^−1^ was used for analyzing the curing behavior.

Tensile properties were measured on dumbbell-shaped samples using an INSTRON 3365 Long travel Elastomeric Extensometer (Norwood, MA, USA) controlled by Bluehill 3 software.

Stress–relaxation experiments were carried out by dynamic mechanical analysis (DMA), using a TA Instruments Q800 (New Castle, DE, USA). Rectangular 36 × 6 × 1.5 mm^3^ samples were initially preloaded at a force of 1 × 10^−3^ N to maintain straightness. After reaching the testing temperature, samples were left for 5 min to reach thermal equilibrium and then strained by 1%, and the deformation was maintained during the test.

## 3. Results and Discussion

### 3.1. Context and Purpose

**Figure 1:**
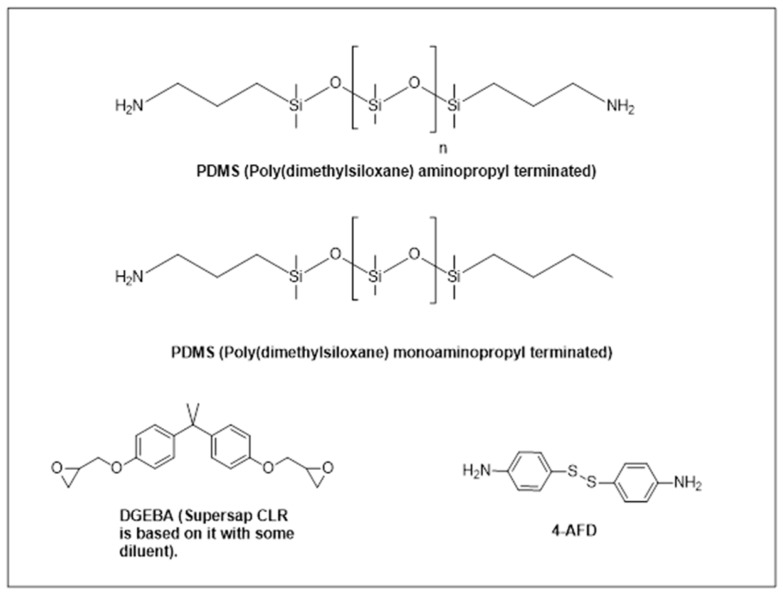
Structure of the used chemicals.

Before presenting the results, it is important to outline the key aspects evaluated in this study. Part of the dynamic amine component in the epoxy formulation was replaced with two different PDMS derivatives, each with two distinct *M*_w_, and tested at different concentrations, aiming to introduce additional surface functionalities while maintaining the intrinsic properties of the 3R network and analyzing the influence of the PDMS structure and concentration. The structures of the used chemicals are shown in Figure 1.

**Figure 2:**
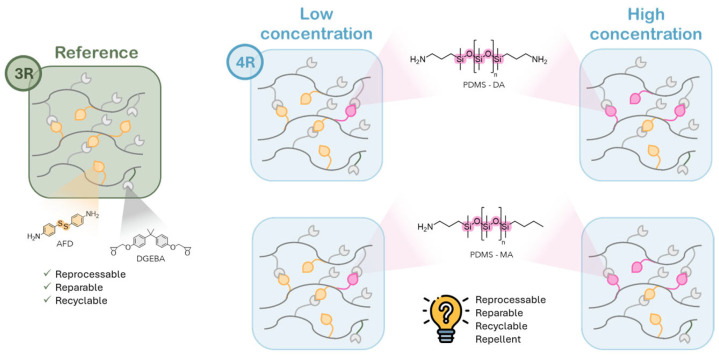
Schematic representation of the work procedure.

Accordingly, we want to verify that the incorporation of PDMS (Figure 2) does not compromise the thermal, mechanical, or dynamic performance of the system, ensuring that recyclability, reprocessability, and repairability were preserved. Additionally, surface properties of the modified resins will be characterized through measurements of water contact angle (WCA), hexadecane contact angle (HCA), water sliding angle (WSA), and hexadecane sliding angle (HSA), parameters that provide quantitative insights into wettability and repellency. Finally, morphological analyses will be performed to correlate the observed surface behavior with structural features of the epoxy matrix. The following sections present and discuss the outcomes of these evaluations.

### 3.2. Characterization of the Thermal Properties

**Figure 3:**
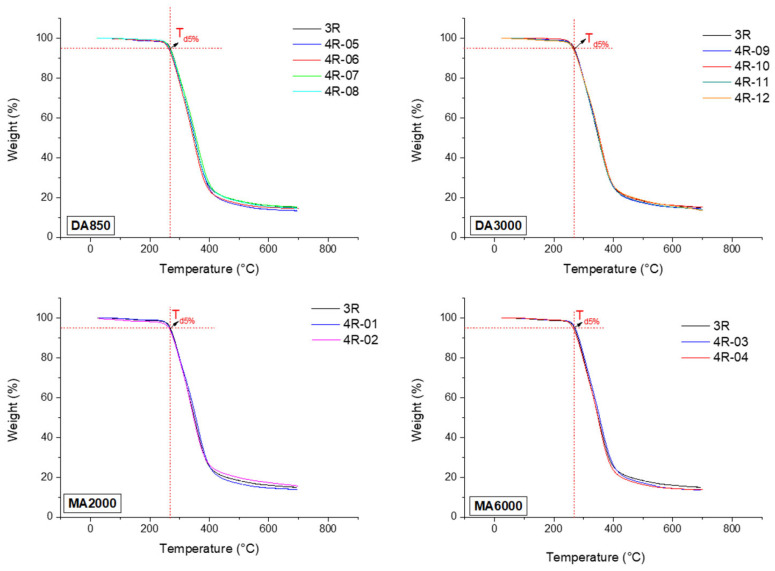
Determination of the degradation temperature of the materials by TGA measurements.

Firstly, thermal stability of the prepared thermoset systems and reactants (Figure S1) was analyzed by TGA. As can be observed in Figure 3, the introduction of PDMS molecules into the 3R resin formulation does not significantly affect the thermal stability of the systems. The T_d5%_ (decomposition temperature corresponding to a 5% weight loss) of the 3R-REF sample was around 268 °C and remained within the same range for all prepared 4R formulations, regardless of the employed PDMS molecule or its concentration. These results indicate that it is possible to incorporate an additional functionality (liquid repellency) into 3R materials without compromising their thermal stability.

To evaluate the effect of PDMS incorporation on the bulk properties of the epoxy networks, the *T_g_* of the different systems was analyzed by DSC (Table 2). The observation of a single thermal transition across the experimental temperature range for all prepared systems, which is attributed to the *T_g_* of the epoxy resin system, indicates that the introduction of PDMS molecules results in uniform and homogeneous materials. A slight decrease in the *T_g_* value was observed in the modified formulations compared to the neat epoxy thermoset 123 °C. This small drop in *T_g_* may be caused by the inherent flexibility of the PDMS chains used as modifiers in the epoxy resin formulation: as PDMS concentration increased, the *T_g_* of the resulting thermoset system decreased due to the higher content of flexible molecules in the formulation. All DSC thermograms are shown in Figure S2.

### 3.3. Surface Properties

Surface wettability was characterized (Table 3) by measuring WCA and HCA, as well as WSA and HSA. These parameters provide insights into the surface energy and adhesion properties of materials. High contact angles (typically > 90°) indicate hydrophobic or oleophobic behavior, while low sliding angles suggest low adhesion and easy droplet mobility [45,46,47]. Together, these measurements help determine the material’s potential for applications requiring self-cleaning, anti-fouling, or low-friction surfaces. All the samples were measured after washing with heptane [48,49].

#### 3.3.1. Static Contact Angle Measurements

As can be observed from the results shown in Table 3, the reference thermoset (3R-REF) already showed quite high static contact angles for water and hexadecane (WCA and HCA, 97° and 35°, respectively). However, neither water nor hexadecane showed any kind of sliding angle on this reference sample.

Then, as previously explained, part of the 4-AFD in the formulation was replaced by different PDMS molecules in various weight percentages to analyze the effect of the PDMS structure and provide the modified formulations with new properties. Two different pathways were analyzed: monoamine-terminated PDMS molecules (with two different *M*_w_, 2000 g·mol^−1^ and 6000 g·mol^−1^) and diamine-terminated PDMS molecules (with two different *M*_w_, 850 g·mol^−1^ and 3000 g·mol^−1^).

On the one hand, when a monoamine-terminated PDMS molecule was used, in any of the analyzed *M*_w_ and at any concentration, WCA increased slightly due to the hydrophobic nature of the PDMS incorporated in the epoxy. It went up to 104° in the case of MA2000 and around 103° in the case of MA6000, indicating an enhancement of the surface hydrophobicity. Moreover, corroborating the improved data, while water did not slide on the reference material, when either MA2000 or MA6000 were introduced in the formulation water showed the ability to slide on the new surfaces. As mentioned before, lower WSA values result in better hydrophobicity and reduced adhesion of water to the surface. Observing the new values, we could affirm that in general, these materials present lower adhesion than the reference. This could be caused by the fact that monoamine-terminated PDMS molecules would be bonded to epoxy molecules just from one end, with their omniphobic end “loose”, favoring the sliding of liquids on their surface [50].

This occurs because PDMS molecules do not swell in water; thus, when water contacts the PDMS layer, a liquid–liquid interface is formed, favoring water sliding [34]. Furthermore, it can be concluded that regardless of the type of monoamine, a higher concentration of PDMS promotes water slippage, as shown by the decrease in WSA when the PDMS concentration increases. When the *M*_w_ increases, the miscibility between water and PDMS decreases; consequently, the formation of a liquid–liquid interface is favored, facilitating the sliding of water droplets off the surface at a low tilting angle [33].

Continuing with the analysis of the samples modified with the monoamine-terminated PDMS, in the case of a non-polar liquid probe as hexadecane, HCA decreased from 35° on reference epoxy resin to 31° and 18° when 4-AFD was replaced by MA6000 (4R-04) and MA2000 (4R-02), respectively. This effect can be attributed to the affinity of PDMS chains for non-polar solvents such as n-hexadecane, which act as good solvents for PDMS. Consequently, the presence of PDMS at the surface promotes increased wetting by hexadecane, shifting the surface properties toward a more oleophilic character [51]. Regarding the HSA, three out of the four samples analyzed when a monoamine-terminated PDMS molecule was added to the 3R formulation, independently of the utilized *M*_w_, did not show any HSA. However, there is a sample, 4R-04 where 0.8% of 4-AFD was replaced by MA6000 where the HSA is 7°. The 4R-04 sample contains the monofunctional molecule with the highest *M*_w_. Due to this high *M*_w_ and the good miscibility of the PDMS molecules with hexadecane, the PDMS molecules swell, which reduces the surface viscosity. Consequently, the sliding resistance of hexadecane on the surface is reduced, and the sample exhibits a low HSA.

On the other hand, when diamin-terminated PDMS molecules were used to replace part of the 4-AFD, as shown in Table 3, the behavior was different. In case of the 4R materials with diamine-terminated PDMS, both WCA and HCA increased, in contrast to materials containing monoamine PDMS, where WCA increased and HCA decreased. This was attributed to the double functionalization of PDMS molecules. The samples 4R-06 and 4R-11 showed the highest WCA, with values that went up to 109° and 111°, respectively. WCA increased due to the hydrophobic nature of the PDMS incorporated in the epoxy. Regarding HCA values, a slight improvement was obtained for the modified thermoset samples, reaching 44° in the case of the 4R-05 and 42° in the case of 4R-11, respectively. It was attributed to double amine functionalization of PDMS molecules, and it does not have an omniphobic end “loose” enough to allow swelling of the PDMS molecules and shift the surface properties toward a more oleophilic character [34].

Regarding WSA, no improvement was obtained for the new thermosets, even at higher concentrations of DA850 and DA3000. This could be explained by the fact that the diamine PDMS molecules are anchored to the epoxy resin by the two ends, leaving less “loose” strings and, thus, less mobility on the surface than in the cases monoamines were used [50]. In the case of HSA, a significant improvement was obtained for HSA: samples containing DA3000 PDMS molecules showed a low sliding angle, HSA < 21°, indicating an enhancement of the surface repellency. In cases where an HSA was determined it could be considered a consequence of the swelling and plasticization of PDMS molecules within the epoxy resin network by n-hexadecane droplets, which significantly increases the mobility of the polymer chains [52].

#### 3.3.2. Dynamic Contact Angle Measurements

The advancing and receding dynamic contact angles (a-WCA, r-WCA) of a drop and the contact angle hysteresis (h-WCA) have been measured. For this purpose, a water droplet is placed on the 4R surface, and the system is tilted. When the water droplet starts sliding, the a-WCA at the front and the r-WCA at the back of the droplet are determined. The difference between them is the h-WCA [15,52]. Wong et al. [53] reported that the sliding angle should decrease with both decreasing liquid surface tension and decreasing h-WCA and showed that other parameters such as roughness, molecular interaction and heterogeneity of surfaces have a big influence on h-WCA. The h-WCA (Table 4) was exclusively determined for samples where a portion of 4-AFD had been substituted with monoamine PDMS because samples containing diamine exhibited no measurable sliding angle (as previously shown in Table 3).

Considering these results, we could conclude that the modified samples exhibited clear hydrophobic behavior, as indicated by their a-WCA between 111° and 117°. In addition, the r-WCA values are relatively high revealing a limited droplet pinning, which reflects good liquid mobility across the surface. Moreover, the hysteresis values obtained indicate moderate adhesion, suggesting that while the surfaces are not fully self-cleaning, they still provide a favorable balance between repellency and droplet stability. At the same time, we must highlight that, notably, the highest *M*_w_ monoamine displayed the lowest h-WCA, which suggests it is the most homogeneous sample [53].

### 3.4. Morphological Analysis

**Figure 4:**
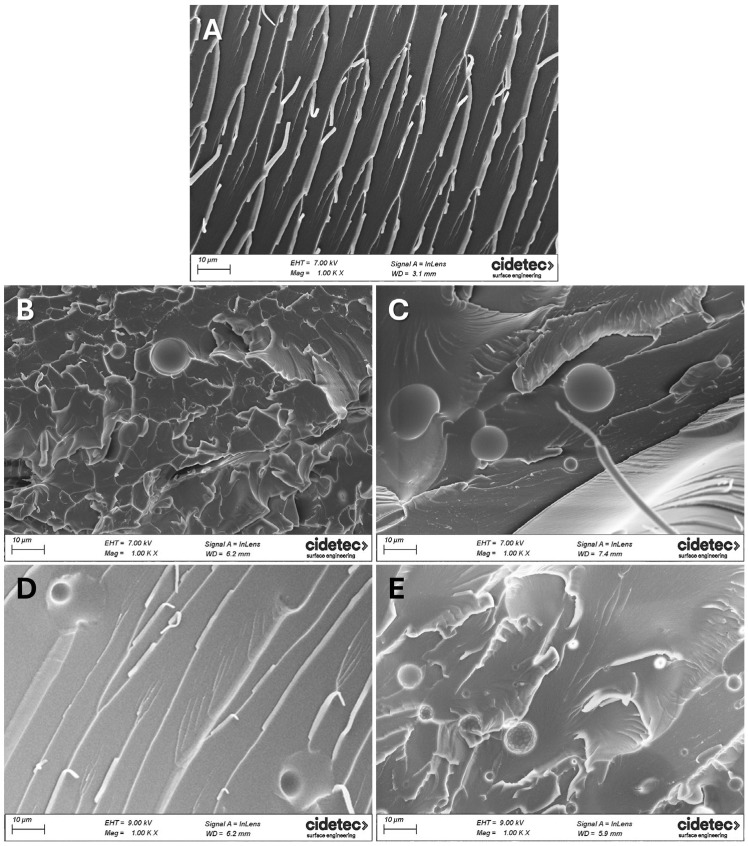
FESEM micrographs of (**A**) 3R unmodified thermoset and different thermosets modified with MA2000 (**B**), MA6000 (**C**), DA850 (**D**) and DA3000 (**E**).

The morphological analyses of fractured surfaces of the thermosets were performed by FESEM. Figure 4 shows the morphology of the fractured surfaces of unmodified and modified thermosets. Figure 4A shows the morphology of 3R-REF, without any PDMS molecule, as a single homogeneous state. Figure 4B,C show the morphology of 4R-02 and 4R-04, respectively. Here, a heterogeneous morphology is observed, with spherical PDMS particles dispersed in the continuous epoxy matrix. EDX analyses of these spheres showed that they were rich in Si, confirming that they were formed by PDMS particles (Figure S3B,C) while the rest of the surface, outside the spheres, showed Si content (Figure S3D).

When the micrographs of 4R-02 and 4R-04 were compared, both containing 0.8%wt PDMS, fewer and smaller spheres were observed when the smaller *M*_w_ PDMS was used. Several works [54,55,56,57] have reported that *M*_w_ is one of the key parameters in the miscibility of different components in a mixture. Usually, when the *M*_w_ increases, the miscibility of substances decreases and as consequence, the diameter and number of spheres increase. The average diameter of the spheres, in our case, was 8.37 ± 5.35 µm for 4R-02 and 17.77 ± 7.25 µm for 4R-04. The increment of sphere-size is attributed to the influence of *M*_w_ on the miscibility of PDMS with epoxy resin.

On the other hand, Figure 4D,E show the morphology of 4R-08 and 4R-12, respectively. Samples containing diamine-terminated PDMS exhibited a similar behavior to the ones containing monoamine-terminated PDMS: two-phase morphology with the epoxy-rich phase forming the continuous matrix and the PDMS phase forming dispersed spherical particles. When the micrographs of 4R-08 and 4R-12 were compared, both containing 3.2%wt of a different diamine-terminated PDMS, it was observed that 4R-08, containing the lower *M*_w_ PDMS, showed fewer and smaller spheres. The average diameter of the spheres was 8.44 ± 2.26 µm and 11.73 ± 5.92 µm for 4R-08 and 4R-12, respectively. As previously explained, the increase in sphere size is attributed to the effect of *M*_w_ on miscibility [54,55,56,57].

### 3.5. Mechanical Properties

The mechanical properties of the modified samples were assessed to ensure they were comparable to those of the reference material (the only expected changes were in the surface properties due to the introduction of additional functionality (such as repellency). The goal was to maintain the original properties, ensuring that the new materials remain suitable for high-performance applications where these properties are essential.

To prove that the 4R formulations with the highest PDMS content were selected and their tensile properties were measured and compared to those of 3R-REF: 4R-02, 4R-04, 4R-08 and 4R-12 were chosen for MA2000, MA6000, DA850 and DA3000, respectively.

A flat laminate of each of these formulations was prepared between two glass plates separated by a 2.5 mm thick rubber, as described in Section 2.2, and their tensile properties were characterized following ISO 527-2 standard [58] with adapted specimen size: dumbbell-type specimens with an overall length (l_3_) of 115 mm; length of narrow parallel-sided portion (l_1_) of 30.0 ± 0.5 mm; distance between broad parallel-sided portions (l_2_) of 58 ± 2 mm; width at ends (b_2_) 10.0 ± 0.5 mm; width at narrow portion (b_1_) 5.0 ± 0.5 mm; thickness (h) 2 ± 0.1 mm; and gauge length (L_0_) 25.0 ± 0.5 mm. Strength-Strain curves are shown in Figure S4.

Results shown in Table 5 prove that the tensile properties and crosslinking density of these thermosets remain in the same range as the reference material, despite the introduction of monoamine/diamine-terminated PDMS molecules in the formulation. In the case of diamines, a slight decrease was observed in the mechanical properties, which can be due to a higher PDMS content being incorporated into the epoxy resin structure when diamine-functionalized PDMS are used, as shown by the morphological analyses. Overall, the obtained values did not exhibit significant variations, indicating that the material remains suitable for these types of applications.

### 3.6. Dynamic Properties

After analyzing and corroborating that the prepared 4R formulations exhibited thermal and mechanical properties comparable to those of the reference 3R formulation, their dynamic properties were measured. First, DMA was used to assess whether dynamic behavior was also preserved in these new formulations. This behavior is governed by the previously mentioned reversible disulfide bonds, which have the capacity to reorganize under specific operating circumstances, enabling the materials to be recyclable, repairable, and reprocessable. Thus, it needs to be demonstrated that the substitution of part of the dynamic hardener by the PDMS molecule that provides the new formulations with repellent properties does not significantly affect this dynamic capacity. To analyze the most extreme situation, we have carried out this analysis on the formulations with the highest amount of PDMS in each case: 0.8%wt for PDMS with a monoamine termination and 3.2%wt for PDMS with a diamine termination.

To assess and compare network dynamics, the most used parameter is relaxation time, defined as the time required for the material to relax 63% of the applied stress. A flat laminate of each of the formulations was prepared between two glass plates separated by a 1.5 mm thick rubber spacer, as described in Section 2.2 and their stress–relaxation behavior was analyzed at 50 °C above the corresponding *T_g_* by DMA (Figure S5).

Based on the results shown in Table 6, it can be concluded that the relaxation time increased slightly upon with the partial substitution of the dynamic hardener by PDMS amines. This increase can be attributed to the reduced network dynamism resulting from the lower content of dynamic hardener in the 4R formulations: modified samples exhibited higher relaxation times (116–168 s) compared to the reference (66 s), consistent with the partial substitution of the dynamic hardener by a non-dynamic component. Although the relaxation process is slower, the values remain within the same order of magnitude, confirming that the networks still retain sufficient dynamic character for the intended applications. New materials have been formulated, which show an additional property (liquid repellency) while maintaining their dynamism and 3R functionality.

Next, the 4R-04 thermoset was selected to corroborate that the higher relaxation times do not significantly affect the materials’ macroscopic performance. This sample was selected because it showed the lowest WSA and the highest relaxation time.

**Figure 5:**
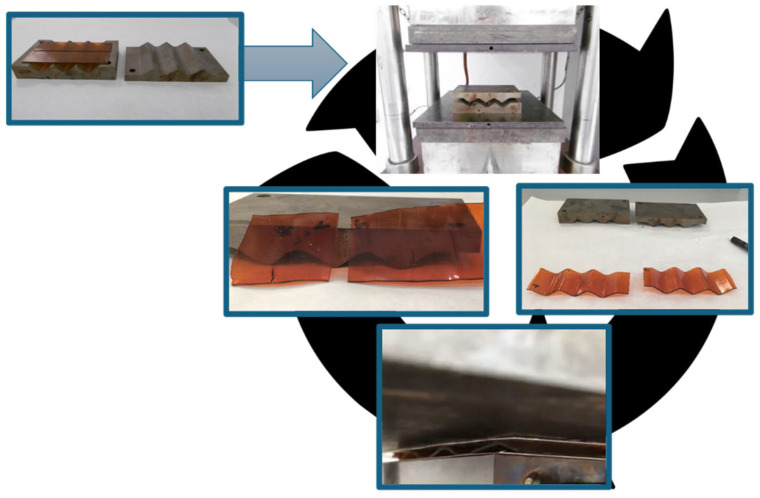
Reprocessing cycle applied to laminates.

Samples 3R-REF and 4R-04 were reprocessed according to the following protocol (Figure 5): a flat thermoset laminate was placed inside a waffled mold and introduced in a hot-press at 170 °C for 1 h. Then, it was allowed to cool down below its *T_g_* inside the press before demolding it to obtain waffled thermoset samples (waffled 3R and waffled 4R, respectively). Then samples were placed again in the hot press for 1 h at 170 °C to flatten them back (flattened 3R and flattened 4R, respectively). Samples were characterized by contact and sliding angles after each step of the process. This procedure allows evaluation of the stability of the functional surface properties after thermal reshaping.

These results demonstrate that 4R thermosets maintain their reprocessability, repairability, and recyclability, indicating that the networks retain sufficient dynamic character for practical use even after multiple processing cycles. To assess whether the surface repellency of the materials is maintained after reprocessing, water and hexadecane contact and sliding angles were measured before and after subjecting the samples to an additional hot-press cycle.

Table 7 presents the surface properties of the laminates throughout the reprocessing cycle. WCA remained essentially unchanged for both samples, while the HCA gradually decreased with each cycle. Sliding angles were consistently absent for the 3R sample, whereas for 4R-04, the WSA decreased, and the HSA disappeared completely. These observations are likely due to changes in surface roughness induced by the repeated pressing cycles, suggesting that the surface repellency is partially affected by reprocessing but remains largely preserved.

To check if the roughness of the samples had changed after the different reprocessing steps and to see if it is the reason for the changes in HSA and WSA, roughness measurements were conducted using confocal microscopy. Figure S6A,B show the confocal images of initial 3R and 4R-04 thermosets, respectively. Initially both samples exhibited a highly wrinkled surface, with Ra values of 0.53 ± 0.11 µm for 3R and 0.39 ± 0.01 µm for 4R thermoset and corresponding Rz values of 3.73 ± 0.76 µm and 2.51 ± 0.17 µm, respectively. Confocal images of 3R and 4R after the reprocessing cycle are shown in Figure S6C,D. The reprocessing led to a significant smoothing of the surfaces, with Ra values of 0.43 ± 0.04 µm and 0.33 ± 0.06 µm and the Rz values of 3.18 ± 0.28 µm and 1.54 ± 0.4 µm, for 3R and 4R, respectively. These results are summarized in Table 8, and it could be affirmed that, as previously noted, surface roughness and heterogeneities play a crucial role in determining surface properties, which explains the observed changes in contact and sliding angles following the reprocessing cycle.

## 4. Conclusions

In summary, a novel thermoset epoxy network that combines high dynamic properties (reprocessability, reparability, and recyclability, 3R) with repellency was successfully developed. This was achieved by replacing part of the 4-AFD with different PDMS molecules containing terminal NH_2_ functional groups, which covalently reacted with the epoxy matrix. The adjustment of the PDMS molecule and its content within the epoxy resin significantly influences the final material’s properties. Our results show that the modified epoxy resin (sample 4R-04), which contained a monoamine-terminated PDMS with a *M*_w_ of 6000 g·mol^−1^, exhibited a low sliding angle to apolar liquids and a diminution of the water sliding angle. Conversely, when the epoxy resin was blended with a diamine-terminated PDMS with a *M*_w_ of 3000 g·mol^−1^ (sample 4R-12), the resulting material not only showed a low sliding angle to apolar liquids but also demonstrated an increase in water repellence.

Furthermore, the experimental results showed that the developed materials maintained their dynamic behavior, preserving the inherent ability to be repaired, reprocessed, or recycled, just as the reference material (3R-REF) did. This enhances their end-of-life treatment possibilities, making such thermosets more competitive and sustainable. The substitution of part of the dynamic hardener (4-AFD) by an amino-terminated PDMS molecule did not have a significant effect on the thermomechanical properties of the material: T_d5%_ remained in the same range (268 °C) for all prepared materials. Although the *T_g_* of the modified formulations was found to be slightly decreased compared with that of the neat epoxy thermoset (set at 123 °C), the mechanical properties were unaffected and remained within the same range as those shown by the 3R-REF.

These findings suggest that the modification of epoxy thermosets with PDMS could enable their broad application in advanced coatings, new matrices for composites, and electronic packaging materials. Furthermore, the inherent ability of these developed materials to be repaired, reprocessed, or recycled enhances their end-of-life treatment possibilities, making such thermosets more competitive and sustainable.

## Figures and Tables

**Table 1:** Code name and characteristics of the PDMS molecules used.

Code Name	Terminal Structure	*M*_w_ (g·mol^−1^)	Amino Equivalent Weight (g·eq^−1^)
MA2000	Monoamine	2000	1000
MA6000	Monoamine	6000	3000
DA850	Diamine	850–900	212.5
DA3000	Diamine	3000	750

**Table 2:** Thermal properties of the prepared formulations.

Sample	PDMS		*T_g_* (°C)
	Molecule	%wt	
3R-REF	-	-	123
4R-01	MA2000	0.2	120
4R-02		0.8	113
4R-03	MA6000	0.2	118
4R-04		0.8	115
4R-05	DA850	0.2	120
4R-06		0.8	114
4R-07		1.6	114
4R-08		3.2	112
4R-09	DA3000	0.2	120
4R-10		0.8	118
4R-11		1.6	117
4R-12		3.2	117

**Table 3:** Surface properties of the prepared formulations.

Sample	PDMS		Surface Properties			
	Molecule	%wt	WCA (°)	HCA (°)	WSA (°)	HSA (°)
3R-REF	-	-	97 ± 3	35 ± 2	DNS	DNS
4R-01	MA2000	0.2	104 ± 1	29 ± 1	70 ± 5	DNS
4R-02		0.8	104 ± 3	18 ± 5	45 ± 5	DNS
4R-03	MA6000	0.2	103 ± 2	30 ± 1	49 ± 6	DNS
4R-04		0.8	100 ± 1	31 ± 1	41 ± 2	7 ± 1
4R-05	DA850	0.2	103 ± 3	44 ± 1	DNS	DNS
4R-06		0.8	109 ± 5	42 ± 1	DNS	DNS
4R-07		1.6	104 ± 2	40 ± 1	DNS	DNS
4R-08		3.2	105 ± 2	41 ± 1	DNS	DNS
4R-09	DA3000	0.2	103 ± 2	39 ± 1	DNS	21 ± 1
4R-10		0.8	109 ± 4	38 ± 1	DNS	15 ± 1
4R-11		1.6	111 ± 2	42 ± 1	DNS	19 ± 4
4R-12		3.2	110 ± 1	45 ± 1	DNS	11 ± 5

DNS: Does not slide. The rate of tilt was 1°·s^−1^ and the tilt range was from 0° to 90°.

**Table 4:** Advancing, receding and hysteresis contact angles for water in some of the samples.

Sample	PDMS		Surface Properties		
	Molecule	%wt	a-WCA (°)	r-WCA (°)	h-WCA (°)
3R-REF	-	-	DNS	DNS	DNS
4R-01	MA2000	0.2	117 ± 4	86 ± 2	31
4R-02		0.8	113 ± 1	83 ± 1	30
4R-03	MA6000	0.2	111 ± 3	87 ± 2	24
4R-04		0.8	112 ± 2	90 ± 1	22

DNS: Does not slide.

**Table 5:** Tensile properties of 3R and 4R formulations.

Sample	PDMS		Tensile Properties			Crosslinking Density *T_g_* + 50 °C
	Molecule	%wt	Fracture Strength (σ_f_) [MPa]	Strain at Fracture Point (ε_f_) [%]	Young’s Modulus (E) [MPa]	Crosslinking Density (ν_XL_) (mol·cm^−3^)
3R-REF	-	-	82 ± 3	5.9 ± 0.8	2952 ± 278	1.7 × 10^−3^
4R-02	MA2000	0.8	80 ± 5	5.7 ± 0.9	3070 ± 393	1.6 × 10^−3^
4R-04	MA6000	0.8	82 ± 2	5.7 ± 0.5	3097 ± 553	1.9 × 10^−3^
4R-08	DA850	3.2	77 ± 2	5.6 ± 0.7	2690 ± 272	1.8 × 10^−3^
4R-12	DA3000	3.2	75 ± 5	4.9 ± 0.9	2686 ± 117	1.7 × 10^−3^

Samples’ dimensions: l_3_ = 115 mm; l_1_ = 30.0 ± 0.5 mm; l_2_ = 58 ± 2 mm; b_2_ = 10.0 ± 0.5 mm; b_1_ = 5.0 ± 0.5 mm; h = 2 ± 0.1 mm; L_0_ = 25.0 ± 0.5 mm.

**Table 6:** Relaxation time of the prepared formulations.

Sample	PDMS		Relaxation Time (s)
	Molecule	%wt	*T_g_* + 50 °C
3R-REF	-	-	66 s (at 175 °C)
4R-02	MA2000	0.8	116 s (at 165 °C)
4R-04	MA6000	0.8	168 s (at 165 °C)
4R-08	DA850	3.2	140 s (at 165 °C)
4R-12	DA3000	3.2	140 s (at 165 °C)

**Table 7:** Surface properties of the 3R and 4R laminate throughout the reprocessing cycle.

Sample	Surface Properties			
	WCA (°)	HCA (°)	WSA (°)	HSA (°)
3R-REF	97 ± 3	35 ± 2	DNS	DNS
4R-04	100 ± 1	31 ± 1	41 ± 2	7 ± 1
WAFFLED 3R	97 ± 2	22 ± 3	DNS	DNS
WAFFLED 4R	98 ± 1	17 ± 2	12 ± 1	DNS
FLATTENED 3R	96 ± 3	17 ± 1	DNS	DNS
FLATTENED 4R	98 ± 2	13 ± 4	17 ± 3	DNS

**Table 8:** Surface roughness of the 3R and 4R laminates throughout the reprocessing cycle.

Sample	Roughness Properties	
	Average Surface Roughness (Ra, µm)	Roughness Depth (Rz, µm)
3R-REF	0.53 ± 0.11	3.73 ± 0.76
4R-04	0.39 ± 0.01	2.51 ± 0.17
WAFFLED 3R	0.43 ± 0.04	3.18 ± 0.28
WAFFLED 4R	0.33 ± 0.06	1.54 ± 0.4

## Data Availability

The original contributions presented in this study are included in the article/Supplementary Material. Further inquiries can be directed at the corresponding author.

## Supplementary Materials

The following supporting information can be downloaded at: https://www.mdpi.com/article/10.3390/polym17233147/s1, Figure S1: TGA curves of the reactants used in 3R and 4R formulations. (a) PDMS molecules and (b) epoxy resin and hardener; Figure S2: DSC curves of neat and modified thermosetting systems, grouped by the PDMS molecule used; Figure S3. FESEM micrograph (top) and EDX analyses (bottom) of 4R-08 thermoset; Figure S4: Strength vs. Strain curves for the 3R and 4R formulations; Figure S5: DMA analyses of the stress relaxation behavior of the prepared formulations; Figure S6: Confocal images of initial 3R (A) and 4R (B) thermosets and of 3R (C) and 4R (D) thermosets after the reprocessing cycle; Table S1: Formulations prepared using the MA2000 PDMS; Table S2: Formulations prepared using the MA6000 PDMS; Table S3: Formulations prepared using the DA850 PDMS; Table S4: Formulations prepared using the DA3000 PDMS.
