# Supplementary material for: Advancing Thermoset Technology: 4R Materials with Unchanged Mechanical Properties and Enhanced Sustainability Through Repellency, Recyclability, Reprocessability, and Repairability
Source: Polymers (Basel). 2025 Nov 26;17(23):3147. doi: 10.3390/polym17233147 (PMC12694553; doi:10.3390/polym17233147)
Supplement: Supplementary file 1 [file polymers-17-03147-s001.zip › polymers-3925538-supplementary.pdf]

Article

Advancing Thermoset Technology: 4R Materials with Unchanged Mechanical Properties and Enhanced Sustainability Through Repellency, Recyclability, Reprocessability, and Repairability

Aratz Genua [1], Nagore Indakoetxea [1], Edurne Elorza [1], Jagoba Iturri [1], Paula Fanlo [1], Elena Jubete [1], Hans-J. Grande [1,2] and Ignacio Garcia [1,*]

[1] CIDETEC, Basque Research and Technology Alliance (BRTA), P° Miramón 196, 20014 Donostia-San Sebastian, Spain; agenua@cidetec.es (A.G.); ejubete@cidetec.es (E.J.)

[2] Advanced Polymers and Materials: Physics, Chemistry and Technology Department Avda, University of the Basque Country (UPV/EHU). Tolosa 72, 20018 Donostia-San Sebastian, Spain

* Correspondence: iggarcia@cidetec.es; Tel.: +34-943309136

Table S1. Formulations prepared using the MA2000 PDMS

Sample	Epikote (g)	Epoxy eq.	PDMS (%wt)	MA2000 (g)	Amine eq.		4-AFD (g)
					MA2000	4-AFD	
4R-01	10.5	0.06	0.2	0,021	$2.1\cdot10^{-05}$	$7.20\cdot10^{-02}$	4.469
4R-02	10.5	0.06	0.8	0.084	$8.4\cdot10^{-05}$	$7.19\cdot10^{-02}$	4.465

Table S2. Formulations prepared using the MA6000 PDMS.

Sample	Epikote (g)	Epoxy eq.	PDMS (%wt)	MA6000 (g)	Amine eq.		4-AFD (g)
					MA6000	4-AFD	
4R-03	10.5	0.06	0.2	0.021	$7\cdot10^{-06}$	$7.20\cdot10^{-02}$	4.470
4R-04	10.5	0.06	0.8	0.084	$2.8\cdot10^{-05}$	$7.20\cdot10^{-02}$	4.469

Table S3. Formulations prepared using the DA850 PDMS.

Sample	Epikote (g)	Epoxy eq.	PDMS (%wt)	DA850 (g)	Amine eq.		4-AFD (g)
					DA850	4-AFD	
4R-05	10.5	0.06	0.2	0.021	$1\cdot10^{-04}$	$7.19\cdot10^{-02}$	4.464
4R-06	10.5	0.06	0.8	0.084	$4\cdot10^{-04}$	$7.16\cdot10^{-02}$	4.446
4R-07	10.5	0.06	1.6	0.168	$8\cdot10^{-04}$	$7.12\cdot10^{-02}$	4.422
4R-08	10.5	0.06	3.2	0.336	$1.6\cdot10^{-03}$	$7.04\cdot10^{-02}$	4.372

Table S4. Formulations prepared using the DA3000 PDMS.

Sample	Epikote (g)	Epoxy eq.	PDMS (%wt)	DA3000 (g)	Amine eq.		4-AFD (g)
					DA3000	4-AFD	
4R-09	10.5	0.06	0.2	0.021	$3\cdot10^{-05}$	$7.20\cdot10^{-02}$	4.469
4R-10	10.5	0.06	0.8	0.084	$1.1\cdot10^{-04}$	$7.19\cdot10^{-02}$	4.464
4R-11	10.5	0.06	1.6	0.168	$2.2\cdot10^{-04}$	$7.18\cdot10^{-02}$	4.457
4R-12	10.5	0.06	3.2	0.336	$4.5\cdot10^{-04}$	$7.16\cdot10^{-02}$	4.443

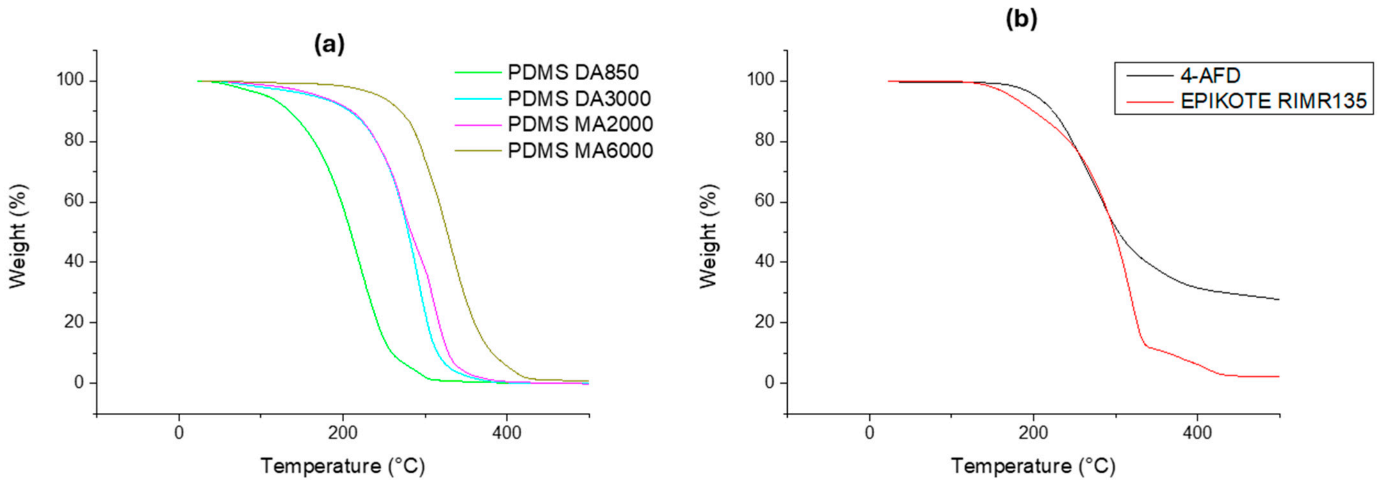

Figure S1. TGA curves of the reactants used in 3R and 4R formulations. a) PDMS molecules and b) epoxy resin and hardener.

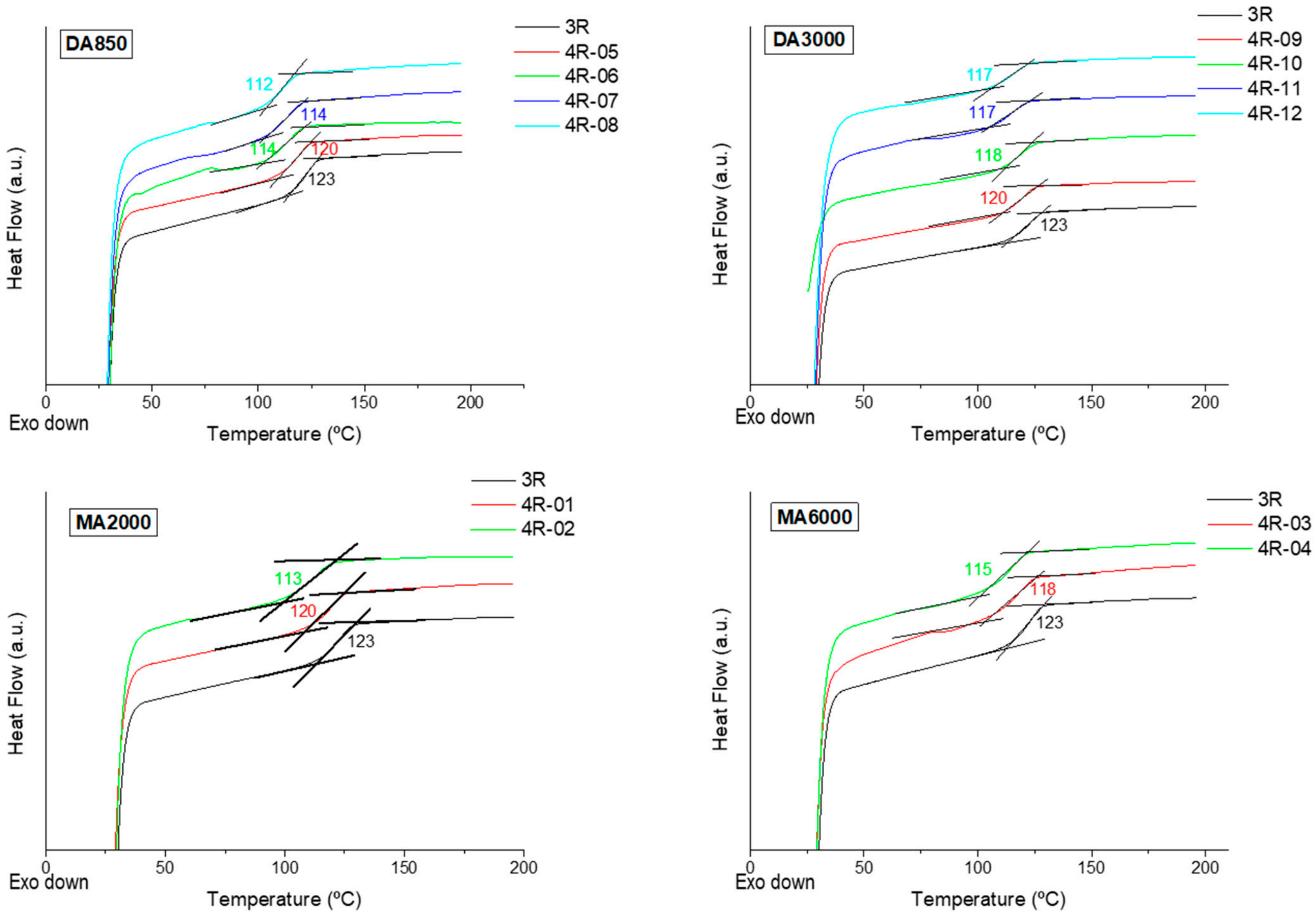

Figure S2. DSC curves of neat and modified thermosetting systems, grouped by the PDMS molecule used.

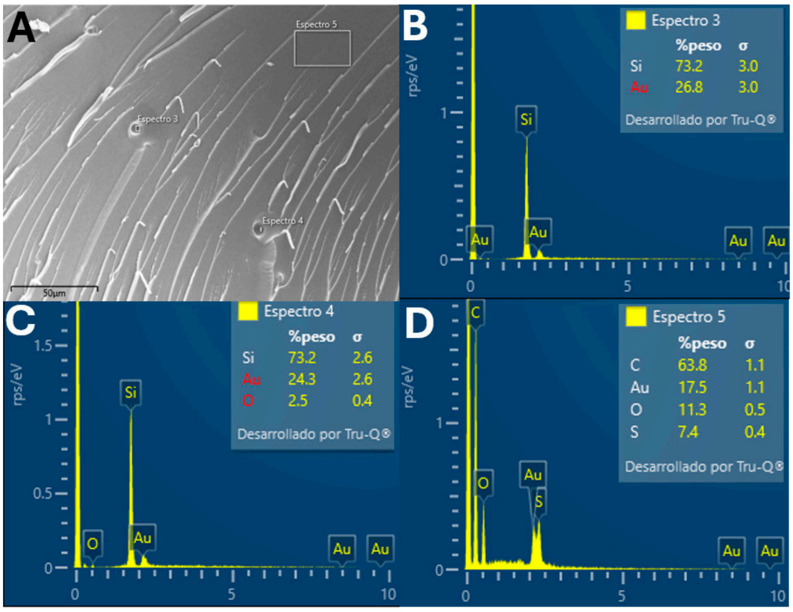

Figure S3. FESEM micrograph (A) and EDX analyses of two zones rich on Si (B and C) and one zone without Si (D) of 4R-08 thermoset.

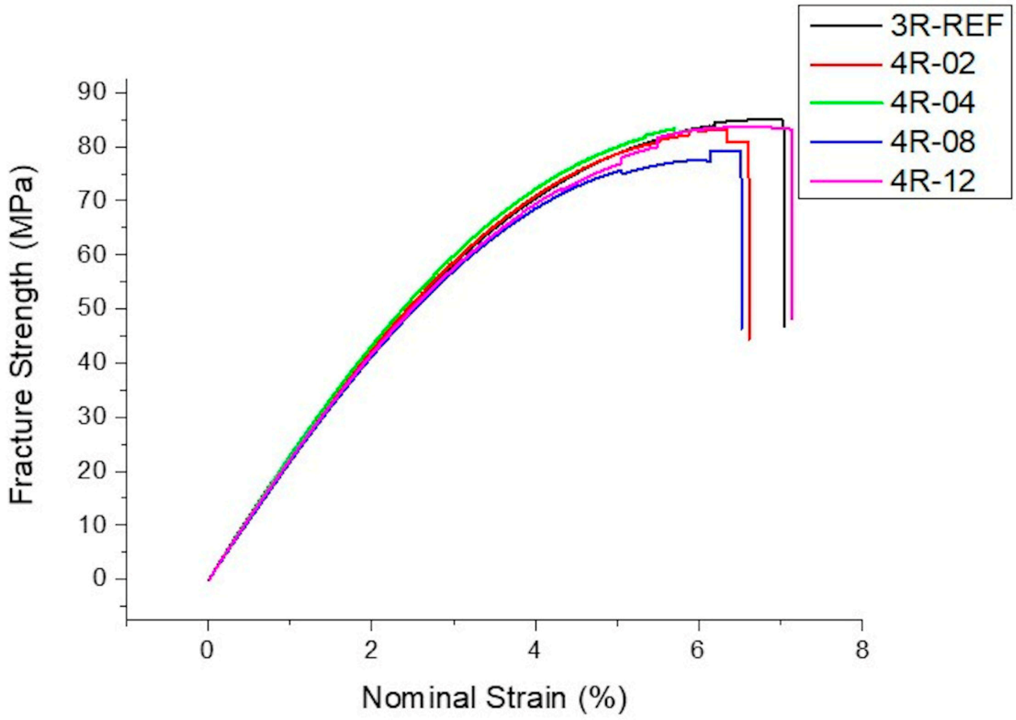

Figure S4. Strength vs Strain curves for the 3R and 4R formulations.

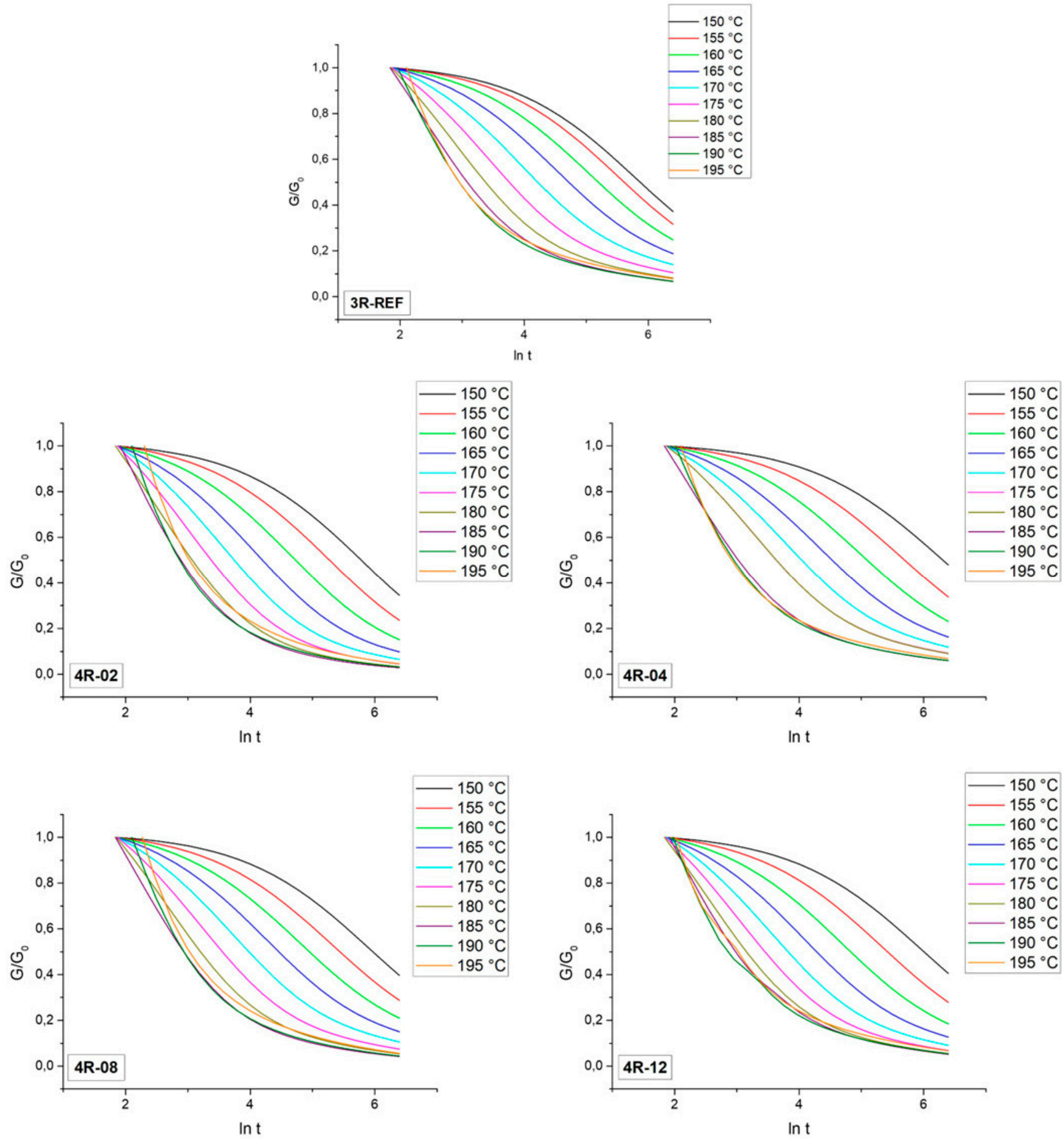

Figure S5. DMA analyses of the stress relaxation behavior of the prepared formulations.

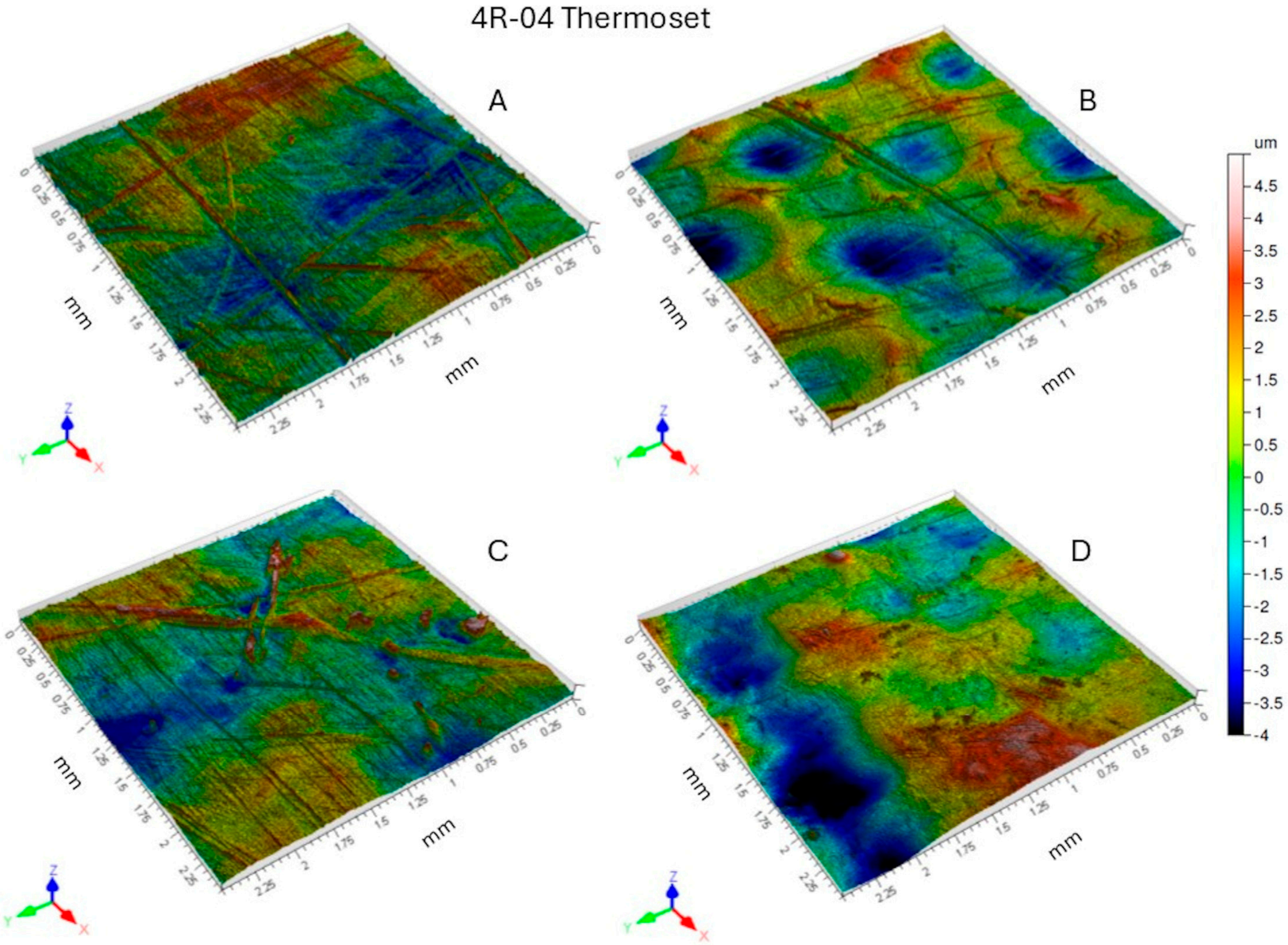

Figures S6. Confocal images of initial 3R (A) and 4R (B) thermosets and of 3R (C) and 4R (D) thermosets after the reprocessing cycle.
